# Tropical understory herbaceous community responds more strongly to hurricane disturbance than to experimental warming

**DOI:** 10.1002/ece3.6589

**Published:** 2020-07-20

**Authors:** Deborah K. Kennard, David Matlaga, Joanne Sharpe, Clay King, Aura M. Alonso‐Rodríguez, Sasha C. Reed, Molly A. Cavaleri, Tana E. Wood

**Affiliations:** ^1^ Colorado Mesa University Grand Junction CO USA; ^2^ Susquehanna University Selinsgrove PA USA; ^3^ Sharplex Services Edgecomb ME USA; ^4^ USDA Forest Service International Institute of Tropical Forestry Río Piedras Puerto Rico USA; ^5^ U.S. Geological Survey Southwest Biological Science Center Moab UT USA; ^6^ College of Forest Resources and Environmental Science Michigan Technological University Houghton MI USA

**Keywords:** climate change, experimental warming, herbaceous, hurricanes, tropical forests

## Abstract

The effects of climate change on tropical forests may have global consequences due to the forests’ high biodiversity and major role in the global carbon cycle. In this study, we document the effects of experimental warming on the abundance and composition of a tropical forest floor herbaceous plant community in the Luquillo Experimental Forest, Puerto Rico. This study was conducted within Tropical Responses to Altered Climate Experiment (TRACE) plots, which use infrared heaters under free‐air, open‐field conditions, to warm understory vegetation and soils + 4°C above nearby control plots. Hurricanes Irma and María damaged the heating infrastructure in the second year of warming, therefore, the study included one pretreatment year, one year of warming, and one year of hurricane response with no warming. We measured percent leaf cover of individual herbaceous species, fern population dynamics, and species richness and diversity within three warmed and three control plots. Results showed that one year of experimental warming did not significantly affect the cover of individual herbaceous species, fern population dynamics, species richness, or species diversity. In contrast, herbaceous cover increased from 20% to 70%, bare ground decreased from 70% to 6%, and species composition shifted pre to posthurricane. The negligible effects of warming may have been due to the short duration of the warming treatment or an understory that is somewhat resistant to higher temperatures. Our results suggest that climate extremes that are predicted to increase with climate change, such as hurricanes and droughts, may cause more abrupt changes in tropical forest understories than longer‐term sustained warming.

## Graphical abstract

We documented the effects of +4°C experimental warming on the abundance and composition of a tropical forest floor herbaceous plant community in Puerto Rico. Results showed that one year of warming did not significantly affect the cover of individual herbaceous species, fern population dynamics, species richness, or species diversity. However, hurricanes Irma and Maria, which hit Puerto Rico after one year of experimental warming, did have significant effects on this understory community.

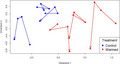

## 1 INTRODUCTION

Changes in the structure, function, and composition of tropical forests in response to climate change may have global consequences due to the forests’ exceptionally high levels of biodiversity and major role in the global carbon cycle (Anderson‐Teixeira, Wang, McGarvey, & LeBauer, 2016; Brodie, Post, & Laurance, 2012; Chazdon et al., 2016; Corlett, 2011; Detwiler & Hall, 1988; Houghton, Byers, & Nassikas, 2015; Pan et al., 2011; Wright, 2013). Nevertheless, despite global models projecting mean temperature increases in the tropics that are either in line with the global mean increase (1.7–3.9°C) or warmer (1.8–5.0°C; A1B Scenario; IPCC, 2013), research addressing the response of tropical ecosystems to climate change lags that of temperate and high‐latitude ecosystems (Corlett, 2012; Wood, Cavaleri, & Reed, 2012). Recent simulation studies of our study site, the Luquillo Experimental Forest in Puerto Rico (USA), predict that future warming and drying may result in large shifts in forest community composition and structure, as well as a transition from a carbon source to a carbon sink (Feng et al., 2018). However, the uncertainties in these simulation studies underscore the need for experiments to isolate and quantify changes to tropical forests due to different types of climate change exposures (Cavaleri, Reed, Smith, & Wood, 2015).

To quantify the effect of increased temperatures on the Luquillo Experimental Forest, the Tropical Responses to Altered Climate Experiment (TRACE) was initiated in 2015 and warming began in 2016 (Kimball et al., 2018). The first tropical forest warming experiment, TRACE uses infrared (IR) heaters under free‐air, open‐field conditions, to warm understory vegetation to 4°C ± 0.1°C and surface soils to 3.3 ± 0.3°C above that of ambient control plots (Kimball et al., 2018). After one year of warming, the study area was strongly affected by the passing of Hurricanes Irma and María, resulting in almost complete defoliation of the forest canopy, broken branches, and downed trees (Reed et al., 2020). This open canopy significantly alters understory microclimate, creating a high light environment with more variable temperature and humidity. Further, during the initial recovery phase throughfall is relatively high, litterfall relatively low, and most of the nutrient uptake is retained by the aboveground vegetation for new tissue production (Scatena, Moya, Estrada, & Chinea, 1996). In the weeks following hurricane disturbance, there is a period of rapid establishment of herbaceous vegetation and seedlings (Comita et al., 2009). Within the context of TRACE, our study addresses the potential for warming to affect the structure and composition of understory herbaceous vascular plants (e.g., ferns, grasses, and forbs), and following hurricane disturbance, we evaluate whether the stress of prior warming affects the trajectories of recovery for the herbaceous community (Johnstone et al., 2016).

Due to the large influence of trees on forest structure and functions, herbaceous plant species that inhabit the forest floor are often overlooked in studies of the effects of climate change on forest dynamics. However, tropical understory plants can approach the diversity of the canopy tree community (Gentry & Dodson, 1987; Linares‐Palomino et al., 2009) and may influence important functions that occur at the forest floor. For example, forest floor herbs provide additional shade and nutrients to the litter layer, modifying abiotic conditions and soil surface habitats for organisms (e.g., invertebrates and fungi) that are vital to decomposition and biogeochemical cycling. In particular, understory fern abundance has been shown to affect seed germination and seedling growth (George & Bazzaz, 1999; Royo & Carson, 2006) and can play a significant role in shaping the trajectory of forest development after disturbances, such as hurricanes (Walker & Sharpe, 2010).

While no previous studies have examined the effects of warming on tropical herbaceous communities, studies do show herbs can be more sensitive to environmental gradients than woody species, which suggests that herbs may also respond to increased temperatures differently than their woody counterparts. For example, tropical understory herbs have been found to have stronger habitat associations related to environmental variables than woody seedlings (Murphy, Salpeter, & Comita, 2016). Herbaceous species have also been found to be more sensitive to drought compared to woody species, a pattern attributed to their shallow rooting depth and lack of secondary tissue (Costa, 2006). Ferns, in particular, can rapidly respond to experimental manipulations (Halleck, Sharpe, & Zou, 2004; Sharpe & Shiels, 2014) and can be sensitive indicators of temperature, humidity, and other microhabitat differences (e.g., Pouteau et al., 2016; Tuomisto & Poulsen, 1996). For example, characteristics of fern leaf emergence events (frequency, presence of spores, leaf length, and number of leaves) were found to rapidly reflect environmental change following hurricane simulations (Sharpe & Shiels, 2014).

Plant communities from higher latitudes vary in their response to warming treatments, but some trends have been identified. In general, climate warming is increasing the dominance of “warm‐adapted” species, or “thermophilization” (De Frenne et al., 2013). Some of the largest warming‐related changes in plant communities are in colder biomes where temperature often limits survival and growth. For example, meta‐analyses of warming studies conducted in tundra describe community shifts that include declining biodiversity and increasing abundance of grasses and shrubs (Walker et al., 2006), but notably these studies also show strong regional variation in plant community responses (Elmendorf et al., 2012). It is unclear if tropical plant communities are more or less sensitive to warming compared to communities at higher latitudes. Plant community responses to warming may be less pronounced in the tropics, where plants are already warm‐adapted. Conversely, tropical communities have had little evolutionary exposure to temperature variability, and therefore, organisms in these environments may be particularly sensitive (Clark, Clark, & Oberbauer, 2010; Malhi, Gardner, Goldsmith, Silman, & Zelazowski, 2014; Williams, Jackson, & Kutzbach, 2007). Warming responses in tropical forest understories may also be attenuated due to dense canopy cover. A meta‐analysis of temperate forests revealed that thermophilization of forest understory plant communities was lower in forests with denser canopies (De Frenne et al., 2013). While there are no published studies on the effects of experimental warming on tropical herbaceous plants, studies using environmental gradients predict that warming may decrease fern abundance and possibly diversity (Pouteau et al., 2016). In terms of interactions with disturbance, the Luquillo Experimental Forest has a long history of hurricane disturbance, which has been found to increase the diversity of the herbaceous community, and thus, the herbaceous community in this forest may have a higher tolerance for variability in light conditions and microclimate than that of other tropical forested ecosystems (Chinea, 1999; López‐Marrero, Heartsill‐Scalley, Rivera‐López, Escalera‐García, & Echevarría‐Ramos, 2019; Meléndez‐Ackerman, Calisto‐Pérez, Morales‐Vargas, & Fumero‐Cabán, 2003).

In this study, we document the effects of experimental warming of +4°C and hurricane disturbance on the abundance and composition of a tropical forest floor herbaceous plant community (ferns, graminoids, forbs, and nonclimbing herbaceous vines) in the Luquillo Experimental Forest, Puerto Rico. We compare changes in (a) fern density, leaf production and mortality, leaf size, and leaf fertility, (b) leaf cover of individual herbaceous species, and (c) species diversity and composition of the herbaceous community over a three‐year period that included one pretreatment year, one year of experimental warming, and one year of recovery following hurricane disturbance.

## 2 METHODS

### 2.1 Study site

**FIGURE 1:**
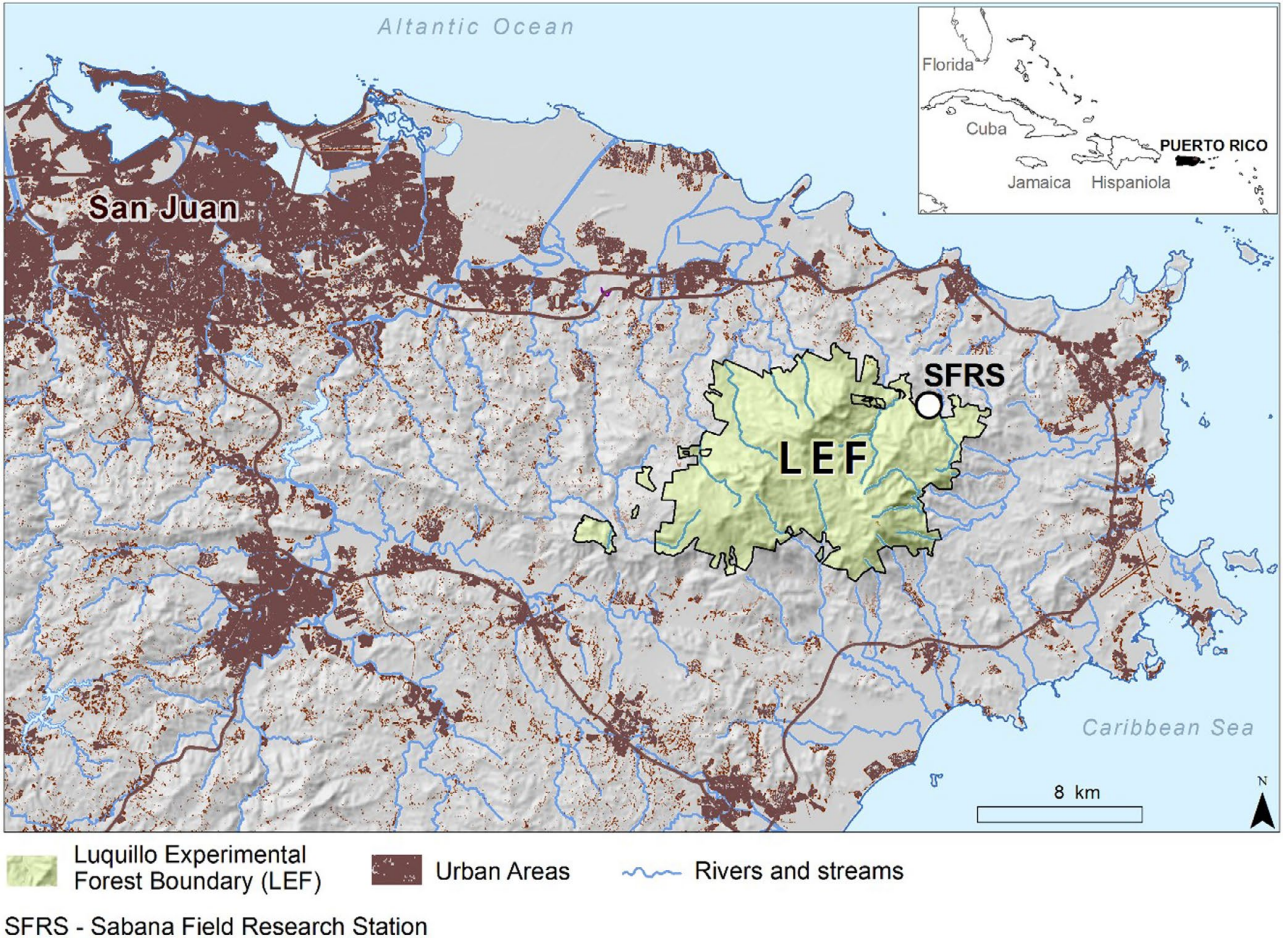
Map showing the study site location at the USDA Forest Service Sabana Field Research Station (SFRS) in the Luquillo Experimental Forest (LEF) in northeastern Puerto Rico

The study takes place in the Luquillo Experimental Forest (LEF) in northeastern Puerto Rico (18°18′N, 65°50′W) near the USDA Forest Service Sabana Field Research Station (Figure 1) at approximately 100 m above sea level (Kimball et al., 2018). The study area is a secondary forest that has regenerated naturally from pasture since the early 1950s (Kimball et al., 2018) and is classified as a subtropical wet forest (Holdridge, 1967). The mean annual precipitation is approximately 3,500 mm per year, although in 2015, Puerto Rico experienced an historic drought with total precipitation measured at El Verde Research Station, approximately 15 km from the study site, approximately 48% lower than the mean of the previous decade (2004–2013; O’Connell, Ruan, & Silver, 2018). Our study began after the acute drought period, which lasted from April–August (O’Connell et al., 2018). In nondrought years, rainfall is highly variable throughout the year with no month receiving less than 200 mm on average (Heartsill‐Scalley, Scatena, Estrada, McDowell, & Lugo, 2007). Mean annual temperature is 24°C and exhibits relatively low seasonal variation, with mean monthly temperature varying just 4°C throughout the year (Kimball et al., 2018). Soils are classified as Ultisols and as such are deep, highly weathered, and contain a high percentage of clay (Scatena, 1989). The site has relatively steep slopes that range from 15 to 26° with an average slope of 21°.

### 2.2 Warming Treatment

Both the control (*n* = 3) and heated (*n* = 3) plots were 4‐m in diameter and hexagonal in shape. In heated plots, three infrared (IR) heaters (Model Raymax 1010, Watlow Electric Manufacturing Co., St. Louis, MO) were installed on cross‐bars at approximately 3.6 m from the ground. All concrete footings, posts, and cross‐bars used for the warming plots were also constructed around control plots to control for effects attributable to treatment installation and shading. The minimum distance separating plots was ~10 m. Warming treatments began September 28, 2016. Heated plots were warmed to maintain a 4.0°C increase in hourly average temperatures to within ±0.1°C compared to unheated plots, as sensed by IR thermometers (Kimball et al., 2018). Heating was relatively homogeneous, with little difference among 0–10 cm depth soil temperatures at the plot centers, edges, and midway between (Kimball et al., 2018). Soil temperatures at the 40–50 cm depth increased about 3°C compared to the controls after a month of warming. Refer to Kimball et al., 2018 for a full description of the warming treatment design, installation, and performance (photos at https://www.forestwarming.org/). Warming treatments were stopped after 11.5 months of heating on September 5, 2017, immediately before hurricane Irma, which passed north of Puerto Rico. Two weeks later, hurricane María struck the island as a category 4 storm with sustained winds up to 250 km/hr and 500 mm of precipitation fell over 24 hr. While the concrete footings, posts, and cross‐bars with the heaters were designed to withstand hurricane force winds, falling trees, and branches during hurricanes Irma and María damaged portions of the heating and plot infrastructure. Tree trunks and large branches were removed from plots following the hurricanes, but leaf litter and small woody debris was kept intact.

### 2.3 Herbaceous plant censuses

#### 2.3.1 Leaf cover

Percent leaf cover of forest floor herbaceous plants (ferns, graminoids, forbs, and nonclimbing herbaceous vines) was estimated in 12 quadrats (0.75 × 0.75 m; 0.56 m^2^) arranged in rows around the center of each plot. Combined, the twelve quadrats covered a contiguous area that represented approximately 65% of each plot. The area omitted from sampling (35% of total plot area) was comprised entirely of plot edges. For the censuses prior to hurricane Irma (2015–2017) a digital image was taken of each quadrat approximately 1 m above the forest floor. Taller nonherbaceous or woody cover (tree saplings) was pulled out of each photo frame. Shorter nonherbaceous cover (tree seedlings) that could not be pulled out of photo frames was digitally “masked” by coloring the green leaf area brown using a digital drawing application (You Doodle, v7.7.5, Digital Ruby, LLC). Total green leaf area of each digital image (masked and unmasked) was then estimated by processing images using Easy Leaf Area (ELA; Easlon & Bloom, 2014). Percentage of the total cover contributed by different herbaceous species was visually estimated for each image and multiplied by the total herbaceous cover to get the percent cover of individual species. Low woody cover (tree seedlings) was estimated as the difference in green leaf area between the unmasked and masked images. Bare ground (no woody or herbaceous cover) was calculated as 100 – total green leaf area (and therefore included leaf litter, woody debris, bare soil, and rocks). Due to the dramatic difference in understory structure pre and posthurricane, our sampling needed to be modified after the hurricanes. Herbaceous cover could not be estimated using digital images in the posthurricane (2018) census due to the vegetation being a relatively tall multi‐layered mat of vegetation. Instead, total percent herbaceous cover, woody cover, bare ground, as well as cover contributed by different species was estimated visually for each quadrat. Percent cover was measured four times over three years: twice before warming treatments began (October 2015 and August 2016), once after 11 months of warming (August 2017), and once 12 months following hurricane passage with no warming (September 2018).

#### 2.3.2 Fern census

In each quadrat, each individual fern leaf >10 cm in length was identified to species, banded, and the petiole length, lamina length, and lamina width were measured. Each measured leaf was classified as fertile (spores present) or sterile (spores absent). In subsequent censuses, surviving and senesced fronds with bands were counted, and new leaves were banded and measured. Ferns were surveyed three times over a two‐year period: a baseline survey 9 months before warming treatments began (October 2015), a pretreatment survey one month before warming treatments began (August 2016), and posttreatment survey after 11 months of warming (August 2017). Leaf production rates were calculated as (new leaves/year)/*N*_0_, where new leaves were the number of new leaves produced per plot over the sampling period, the sampling period (year) calculated as the number of sampling period days/365, and *N*_0_ was the initial starting population per plot for that time period. Leaf mortality rates were calculated similarly (leaves died/year)/*N*_0_). Due to dense understory vegetation posthurricane, the fern survey was not conducted in 2018 because locating ferns below the litter and new vegetation layers would cause too much disturbance to the vegetation structure and affect other components of the TRACE study.

### 2.4 Statistical analysis

Repeated measures analysis of variance (ANOVA) tests were used to examine treatment effects (warming vs. control) and differences over time for herbaceous cover, woody cover, bare ground, cover of the four most common herbaceous species, species richness, and species diversity (Simpson's Index). Repeated measures ANOVAs were also used to examine treatment effects and differences over time for leaf counts, leaf lengths, leaf production rates, leaf mortality rates, and proportion of fertile leaves of *Blechnum occidentale*. The proportion of fertile fern leaves was arcsine transformed prior to analysis. Two other fern species (*Adiantum latifolium* and *Thelypteris deltoidea*) occurred in only two plots each and therefore were not analyzed statistically. We used nonmetric multidimensional scaling (NMDS) to explore patterns in vegetation composition over time and between treatments. Nonmetric multidimensional scaling is a nonparametric ordination method for assessing similarities between objects (e.g., plant communities) in a relatively low‐dimensional space (Kruskal, 1964). We used the R package vegan (Oksanen et al., 2015) for the NMDS analysis and used the standard distance metric and settings recommended by the package authors (e.g., the Bray‐Curtis distance; Bray & Curtis, 1957).

## 3 RESULTS

### 3.1 Herbaceous cover

**FIGURE 2:**
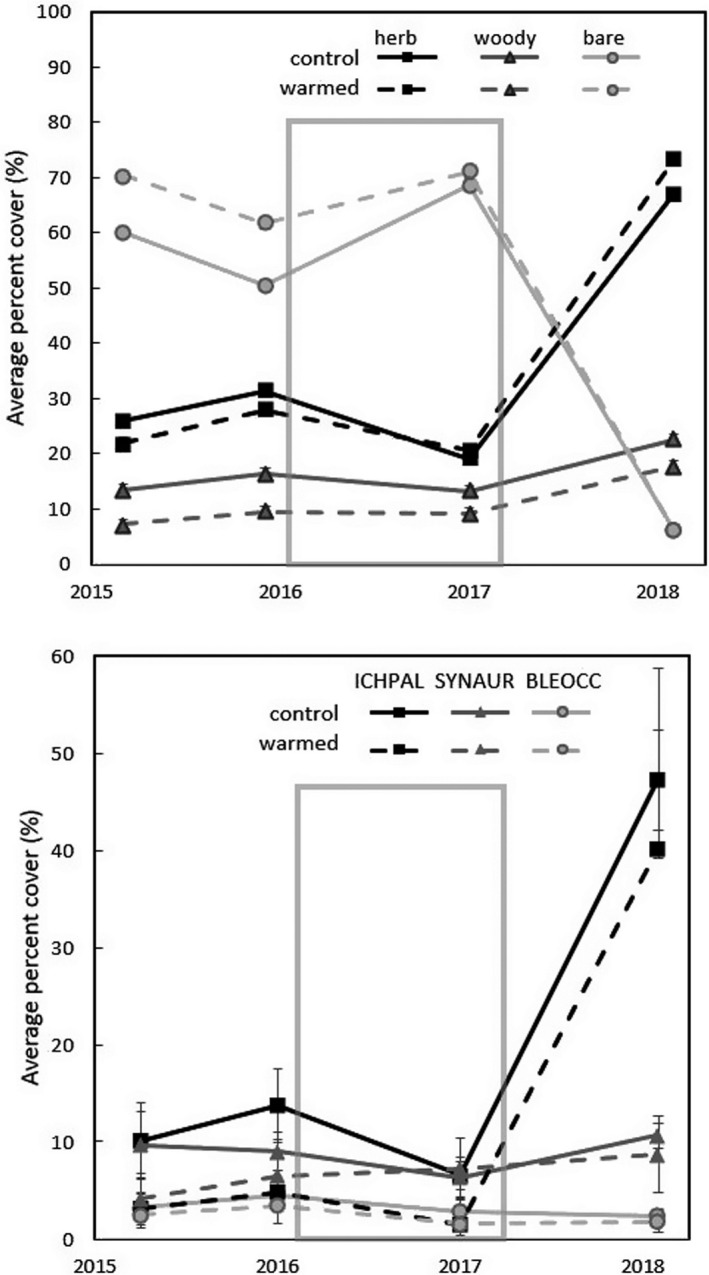
Average percent cover of A) herbaceous species (herb), tree and liana seedlings (woody), and nonplant cover (bare) and B) the most abundant grass (ICHPAL = *Ichnanthus pallens*), forb (SYNAUR = *Syngonium*), and fern (*Blechnum occidentale* = BLEOCC) species in control and experimentally warmed plots (*n* = 3) sampled once a year for four years. Years on the x‐axis represent the following sampling months: October 2015 (baseline survey), July 2016 (prewarming), August 2017 (1 year postwarming), and Sept. 2018 (1 year posthurricane). The gray box shows the period of time the warming treatment was active before it was stopped due to damage by two hurricanes in September 2017. (Error bars ± 1 *SE*)

The warming treatment did not affect total herbaceous cover over the study duration (*p* = .99). Herbaceous cover did change significantly over time (*p* < .0001), increasing more than threefold after the hurricanes, from 19% to 67% in control plots and from 20% to 73% in warmed plots (Figure 2a). Most of this increase in herbaceous cover was due to the increase in abundance of one graminoid species (*Ichnanthus pallens*), which significantly increased after the hurricanes (*p* < .001) to comprise 71% and 55% of the total herbaceous cover in the control and warmed plots, respectively (Figure 2b). There was no warming effect on the cover of *I. pallens*, however (*p* = .36). The second and third most abundant herbaceous species (the nonclimbing vine *Syngonium* sp. and the grass *Pharus* sp.) did not differ between warming treatments (*p* = .52; *p* = .33) or over time (*p* = .51, *p* = .92).

Bare ground decreased significantly over time (*p* < .0001) from an average of 70% before the hurricanes to an average of 6% after the hurricanes. Most of this decrease in bare ground was due to the increase in herbaceous cover rather than woody cover (Figure 2a). Woody cover increased significantly after the hurricanes (*p* = .002), although to a lesser degree than herbaceous cover. Warming did not have a significant effect on either bare ground (*p* = .35) or woody cover (*p* = .16).

### 3.2 Fern abundance, size, fertility, and mortality rates

**FIGURE 3:**
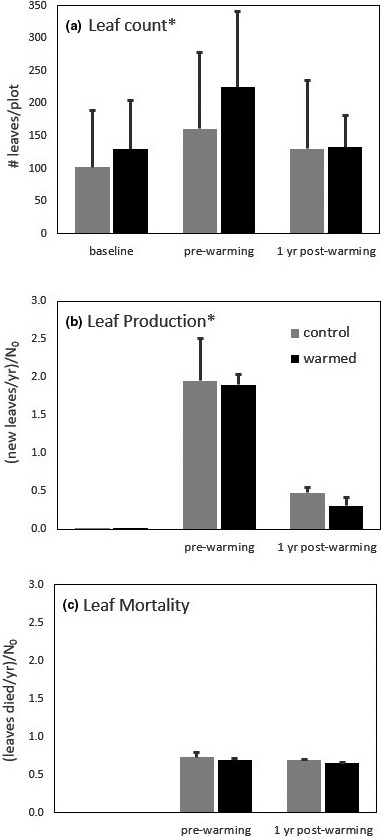
Population dynamics and size of *Blechnum occidentale* prior to and 1 year postwarming. A) Average number of leaves alive in each plot at each survey, B) average number of new leaves produced annually as a proportion of the initial population size (N_o_), and C) average number of tagged leaves that died annually as a proportion of the initial population size (N_o_). The baseline survey was conducted October 2015, the before warming survey in July 2016, and 1 year postwarming survey in August 2017. *B. occidentale* was not found in one warmed plot, therefore *n* = 3 for the control and *n* = 2 for the warmed treatment. No parameters were significantly different between treatments. Parameters that varied significantly over time (*p* < .05) are indicated by an asterisk. (Error bars ± 1 *SD*)

Warming did not affect the cover (Figure 2b), leaf counts (Figure 3a), or leaf production rates (Figure 3b) of *B. occidentale* (*p* = .65; *p* = .75, *p* = .62, respectively); however, these parameters did change significantly over time (*p* = .01, *p* = .03, *p* = .007), with leaf cover and count peaking in August 2016 before the warming treatment started, and leaf production higher during the prewarming period. The change in leaf abundance during the study period was largely driven by leaf production rather than mortality. Leaf mortality (Figure 3c) and leaf length (data not shown) of *B. occidentale* did not differ between treatments (*p* = .25, *p* = .16, respectively) or over time (*p* = .18, *p* = .88). Warming did not affect the percentage of new *B. occidentale* leaves that were fertile (*p* = .35). The percentage of fertile leaves did change significantly over time (*p* = .03) peaking at 11% in August 2016, before the warming treatment began. The percentage of fertile leaves did not exceed 2% at any other census.

### 3.3 Diversity and composition

**FIGURE 4:**
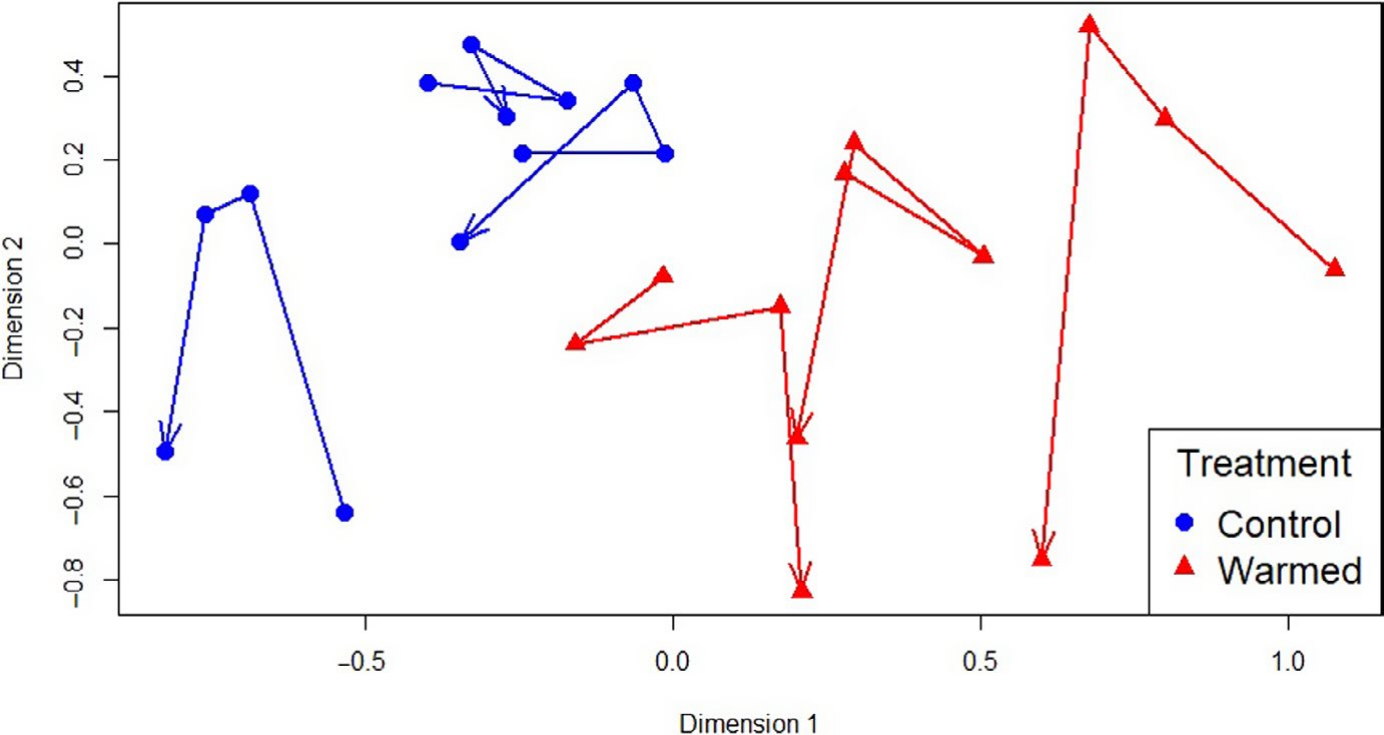
Nonmetric dimensional scaling analysis showing similarities in herbaceous species composition between the control (*n* = 3, in blue) and warmed (*n* = 3, in red) plots over four years (2015– 2018), with one data point per plot per year. Lines connect values from a single plot in chronological order and display the individual trends over time. Points that are closer together represent plots and years that had more similar species composition. The first point in each line represents October 2015 (baseline survey), the second point July 2016 (prewarming), the third point August 2017 (1 year postwarming), and the last point September 2018 (1 year posthurricane)

Neither species richness nor the Simpson's diversity index differed between the control and warmed plots (*p* = .11, *p* = .28, respectively) or over time (*p* = .22, *p* = .33). The NMDS model with the typical two‐dimensional ordination was a good fit to the data with a computed stress value of 0.16 (where < 0.25 is considered a good fit). The NMDS plot revealed that the plant communities of the controlled and warmed plots were different throughout the study, from prewarming to posthurricane (Figure 4). The largest shift in plant community composition was after the hurricane, in warmed plots.

## 4 DISCUSSION

The goal of this study was to determine if +4°C experimental warming would affect the abundance and composition of the herbaceous component of a tropical forest understory. We found that warming had negligible effects on the herbaceous community after one year of warming. While we intended to monitor the effects of warming for multiple years, infrastructure damage caused by hurricanes Irma and María halted the warming treatment after one year. These hurricanes caused dramatic effects on the herbaceous community, consistent with what has been reported for other functional groups following other hurricanes in Puerto Rico (Brokaw & Grear, 1991; Brokaw & Walker, 1991; Lugo, 2008; Walker, Lodge, Guzman‐Grajales, & Fetcher, 2003; Zimmerman et al., 1994), and offered the opportunity to compare the effects of warming with those from hurricanes.

We found no significant effect of one year of warming on the understory herbaceous community. The short duration of the warming treatment makes it difficult to infer longer‐term shifts in tropical forest understory plants. Nevertheless, due to their relatively fast leaf turnover compared to woody species, herbaceous species are capable of dramatic changes over short time spans and therefore the lack of change found after one year of warming may be reflective of an understory that is somewhat resistant to higher temperatures. Earlier studies of ferns in Puerto Rico have found they can rapidly shift their species‐specific rates of leaf production, leaf size, and fertility within a year after experiencing changes in environmental conditions (Sharpe, 2010; Sharpe & Shiels, 2014). During this study, the high leaf counts and production rates of *B. occidentale* during the pretreatment period may have been a recovery response to the end of the 2015 drought. If true, this response highlights even more the resistance of this fern species to warming as compared to drought stress.

Warming experiments in temperate systems have found significant changes in plants after similarly short treatment periods. For example, experimental warming of tallgrass prairie in Oklahoma significantly increased aboveground green biomass of grasses after only 6 months of warming (Wan, Hui, Wallace, & Luo, 2005). A meta‐analysis of warming experiments in tundra detected changes in cover of shrubs, grasses, moss and lichen and plant diversity with experimental warming of 1–3°C after two years, which would represent less than twelve months of growing season (Walker et al., 2006).

The relative resistance of the herbaceous understory to one year of warming may be related to the low light conditions at the forest floor. De Frenne et al. (2015) experimentally showed that tree shade slowed changes related to warming treatments in forest understories and concluded that light limitation was a likely explanation for the slow biotic responses to warming observed in temperate forest understories (De Frenne et al., 2013). The previously mentioned studies that reported rapid responses to experimental warming are in biomes that lack an overstory and are less light limited (tundra and grasslands). Light is one of the most limiting resources for plant growth in wet tropical forests (Denslow, 1987), and therefore, canopy shading may produce even more of a lag effect of warming in tropical forests. Before hurricane passage, TRACE plots were under continuous forest canopy and therefore experienced these very low light conditions typical of tropical forest understories.

Even though warming responses were not observed after one year, it is possible that the herbaceous community would change over longer time periods. Using elevational gradients in Tahiti, Pouteau et al. (2016) showed that fern cover decreased with increasing temperature and fern diversity varied in association with temperature, a pattern that suggests warming could change fern abundance and diversity over longer time scales.

The changes caused by hurricanes Irma and María dramatically increased herbaceous cover; however, this was mostly due to increased cover of one grass species (*I. pallens*). This response is somewhat consistent with observations following a canopy trimming experiment in nearby Luquillo Experimental Forest (Puerto Rico) that found graminoid cover to increase 10‐ to 20‐fold after canopy removal treatments (Shiels, Gonzalez, Lodge, Willig, & Zimmerman, 2015). The other grass species found in TRACE plots (*Pharus* spp.) did not increase after the hurricanes, suggesting not all grass species will respond to hurricanes similarly, but rather according to their shade‐tolerances. The response among the fern species was also mixed. The most common fern species peaked in cover during the prewarming year but did not change during the warming treatment or after the hurricanes. This response is different from observations following the canopy trimming experiment in the Luquillo Experimental Forest, where Sharpe and Shiels (2014) found increased leaf number and size of two common fern species (*Cyathea borinquena* and *Thelypteris deltoidea*) in response to canopy opening. Again, these contrasting patterns may be due to differences in fern species’ shade‐tolerances.

Ferns have low resistance to trampling, and due to the small size of the TRACE plots, even light trampling by researchers may have affected the results. Halleck et al. (2004) estimated that changes to vegetation structure and mortality from incidental trampling by researchers resulted in up to a 50% reduction of fern abundance over a six‐year period in a study located in nearby Luquillo Experimental Forest. However, the effects of trampling should have been consistent between the control and warming plots and is therefore not likely to have affected detecting the effects of warming.

## 5 IMPLICATIONS AND CONCLUSIONS

Projected temperature increases in Puerto Rico are lower than those projected globally or for the tropics, with a temperature increase of 1.51–1.75°C projected for 2081–2100 (RCP6.0, increase relative to 1986–2005; Harter et al., 2015). The lack of an effect caused by more than double this amount of warming, as seen in our study, may indicate that temperature increases will not be the most consequential component of climate change for these understory herbaceous communities, at least over the short term. Along with the gradual increase in temperature, climate change is also predicted to increase the frequency of climate extremes, such as droughts (IPCC, 2013; Trenberth et al., 2014), heat waves, hurricanes, and other heavy wind and precipitation events (Fisher & Knutti, 2014; IPCC, 2013). These climate extremes are likely to cause more abrupt changes in structure, function, and composition of forests than the gradual increase in mean temperature (Dale et al., 2001). As we observed, the disturbance caused by hurricanes had dramatically greater short‐term effects on the understory plant community than increased temperatures. While there is uncertainty about the future frequency of hurricanes, there is a consensus that the intensity of hurricanes will increase 2%–11% by 2100 (Knutson et al., 2010). While the Luquillo forest is generally thought to be highly resilient to hurricane disturbance (Brokaw et al., 2012), it is unknown how increased hurricane intensity might change the response of this forest ecosystem, or how warming could interact with hurricane disturbance to affect tropical forest understories. This question can be asked as the warming treatments continue.

Of the climate extremes expected to increase, Frank et al. (2015) suggest droughts have the strongest and most widespread effects on terrestrial carbon cycling. Drought experiments in tropical forests have shown decreased tree growth, increased tree mortality, and changed species composition (Corlett, 2016). However, there is evidence to suggest that plant species in the Caribbean may have evolved under drought stress and possess traits that contribute to a degree of drought resistance (Borhidi, 1985; Lugo, Medina, Cuevas, & González, 2019). In Puerto Rico, precipitation is projected to decrease by 10.0%–15.6% by 2081–2100 (RCP6.0, increase relative to 1986–2005; Harter et al., 2015). And, similar to many mountainous tropical islands, shifts in the trade‐wind inversion layer may be one of the more consequential effects of climate change on Puerto Rico by affecting orographic cloud formation (and thus rainfall, solar radiation, temperature, and humidity patterns; Harter et al., 2015). This effect could be especially significant on the windward side of Puerto Rico, where this study was located. Given the history of hurricane disturbance in these ecosystems, the herbaceous communities may be more resistant to variability in microclimate brought about by these potential climatic changes.

Regardless of which aspect of climate change has the greatest effect on plant communities in Puerto Rico, results from this study suggest the understory herbaceous community is relatively resistant to changes in temperature over the short term. The large shift in leaf cover, and smaller shift in species composition, that was observed following hurricane disturbance indicates light availability or other consequences of hurricane‐induced changes to the forest exert a stronger control over the herbaceous community than temperature. Considering both tropical forests and islands harbor, a disproportionately high fraction of global biodiversity, continued study of the effects of climate change on tropical forests, particularly on islands, is critical.

## CONFLICT OF INTEREST

None of the authors has any competing interests.

## AUTHOR CONTRIBUTIONS

**Deborah K. Kennard:** Data curation (lead); Formal analysis (equal); Investigation (lead); Methodology (lead); Writing‐original draft (lead); Writing‐review & editing (lead). **David Matlaga:** Data curation (supporting); Formal analysis (equal); Investigation (supporting); Methodology (supporting); Writing‐original draft (supporting); Writing‐review & editing (supporting). **Joanne Sharpe:** Data curation (supporting); Investigation (supporting); Methodology (supporting); Writing‐original draft (supporting); Writing‐review & editing (supporting). **Clay C. King:** Formal analysis (equal); Writing‐review & editing (supporting). **Aura M. Alonso‐Rodríguez:** Investigation (supporting); Project administration (supporting); Writing‐review & editing (supporting). **Sasha C. Reed:** Conceptualization (equal); Funding acquisition (equal); Writing‐review & editing (supporting). **Molly A. Cavaleri:** Conceptualization (equal); Funding acquisition (equal); Writing‐review & editing (supporting). **Tana E. Wood:** Conceptualization (equal); Funding acquisition (equal); Project administration (lead); Writing‐review & editing (supporting).

## Data Availability

Data are archived and publicly accessible in Dryad (https://doi.org/10.5061/dryad.f1vhhmgtr).
